# Four-year Outcomes of Class III and IV Anterior Restorations Based on a Subset of German Health Insurance Data

**DOI:** 10.3290/j.jad.b5733812

**Published:** 2024-09-04

**Authors:** Michael Raedel, Susann Hertel, Heinz-Werner Priess, Aikaterini Mikeli, Vadim Kopzon, Steffen Bohm, Michael H. Walter

**Affiliations:** a Dentist, Senior Lecturer, Group Leader Health Service Research, Prosthodontics, Carl Gustav Carus Faculty of Medicine, Technische Universitaet Dresden, Fetscherstraße 74, 01307 Dresden, Germany. Conceptual study design, data interpretation, manuscript draft.; b Dentist, Specialist in Pediatric Dentistry, Operative and Pediatric Dentistry, Carl Gustav Carus Faculty of Medicine, Technische Universitaet Dresden, Fetscherstraße 74, 01307 Dresden, Germany. Data interpretation, manuscript draft, reviewing literature.; c Bio-Statistician, Group Leader Statistical Analyses, AGENON Gesellschaft für Unternehmensentwicklung im Gesundheitswesen, Friedrichstraße 94, 10117 Berlin, Germany. Conceptual study design, data analysis, statistical evaluation.; d Dentist, Specialist in Prosthetic Dentistry, Prosthodontics, Carl Gustav Carus Faculty of Medicine, Technische Universitaet Dresden, Fetscherstraße 74, 01307 Dresden, Germany. Data interpretation, manuscript draft.; e Dentist, Specialist in Oral Surgery, Prosthodontics, Carl Gustav Carus Faculty of Medicine, Technische Universitaet Dresden, Fetscherstraße 74, 01307 Dresden, Germany. Data interpretation, manuscript draft.; f Managing Director (Research), AGENON Gesellschaft für Unternehmensentwicklung im Gesundheitswesen, Friedrichstraße 94, 10117 Berlin, Germany. Conceptual study design, data analysis, data interpretation.; g Dentist, Senior Lecturer, Former Head of Department, Prosthodontics, Carl Gustav Carus Faculty of Medicine, Technische Universitaet Dresden, Fetscherstraße 74, 01307 Dresden, Germany. Study supervisor, conceptual study design, data interpretation, manuscript draft.; * both authors contributed equally to the manuscript

**Keywords:** anterior restoration, outcome, public health, dental general practice, survival analysis

## Abstract

**Purpose:**

Numerous studies report on the outcome performance of posterior composite restorations. However, there are fewer studies providing data for anterior restorations. The aim of this study was to evaluate the clinical outcome performance of anterior permanent restorations by analyzing a large dataset from a German national health insurance company.

**Materials and Methods:**

Routine claims data from a major German national health insurance company were assessed. Fee codes were used for tracing restoration careers on a day-count basis. The treatment was defined as a placed restoration (Class III and IV) on a mesial or distal tooth surface, irrespective of the extension. The restorations were placed between January 1, 2010 and December 31, 2013. Statistical analyses were conducted using Kaplan–Meier survival analysis to determine cumulative 4-year survival rates. The primary outcome was re-intervention on the same surface. Secondary outcomes were crowning and extraction which were analyzed separately.

**Results:**

A total of 2,417,791 restorations involving mesial surfaces and a number of 2,409,031 restorations involving distal surfaces were observed. At 4 years, the cumulative survival rates concerning the primary outcome “re-intervention” were 79.9% for mesial and 80.9% for distal restorations. The respective annual failure rates (AFR) were 5.5% and 5.2%. Four-year survival rates for the secondary outcome “crown” were 93.8% for mesial and 94.1% for distal anterior restorations. The respective AFRs were 1.6% and 1.5%. For the secondary outcome “extraction,” the respective rates were 94.6% for mesial and 93.9% for distal restorations. The respective AFRs were 1.4% and 1.6%.

**Conclusion:**

The performance of permanent anterior restorations which were placed in general dental practices in Germany can be rated as acceptable.

Despite the decline in dental caries over the last decades, there is still a high demand for restorative procedures in clinical dental practice.^10^ Cost- and time-effective chair-side restorations play an essential role. Because esthetics are the primary goal in the anterior region, composite resin combined with bonding techniques is the material of choice.^20^ Numerous clinical studies and data on the performance of posterior composite restorations are available.^1,8,24^ In a recent systematic review that evaluated the longevity of posterior restorations according to material and adhesive class, it was found that the survival rate of posterior composite restorations decreased to approximately 85–90% after 10 years, with no significant difference between hybrid, microhybrid, and nanohybrid resin materials.^15^

Anterior restorations however have a different failure pattern compared to posterior restorations.^15^ Data on the long-term longevity and clinical performance of anterior restorations are still limited.^11,19^ However, there are five major studies addressing the long-term survival of anterior restorations.^5,7,11,16,25^ A systematic review by Demarco et al included 17 studies that investigated the clinical survival of Class III and IV composite restorations in the permanent dentition. Both prospective and retrospective studies were included.

The follow-up periods ranged from 3 to 17 years, and the number of restorations differed between 25 and 341. Annual failure rates (AFR) varied from 0% to 4%.11 In the same year, Heintze et al published a review and meta-analysis of the clinical effectiveness of direct anterior restorations.^16^ However, the working group only included prospective clinical studies with a minimum observation period of 2 years; retrospective studies were excluded. In addition, both chemically cured composites and resin-modified glass-ionomer cements were included. This study found 10-year survival rates of 95% for Class III restorations and 90% for Class IV restorations, with an AFR between 0.5% and 1%. These results are in agreement with those of Demarco et al.^11,16^

A recent systematic review of long-term survival of anterior restorations by Shah et al included the study by Demarco et al and 11 other studies published between 2011 and 2018.^25^ The AFR ranged from 0% to 27.11% and survival rates varied from zero to 100%. However, the high AFR of 27.11% compared to previous publications is mainly due to a study by Gulamali et al in which patients with localized anterior wear were treated with Dahl composite restorations to increase the vertical dimension.^14,25^

However, most long-term studies have been conducted in university settings. In addition, different study criteria are used to assess the failure or success of filling longevity, such as the USPHS criteria or modified USPHS FDI criteria, and some studies did not specify the assessment method.^11,16,17,25^

An objective method to gain information on the outcome of a dental treatment is the use of insurances’ databases or public health services. Collares et al conducted an analysis of electronic health record data from 24 general dental practices in the Netherlands and retrospectively examined the longevity of 72,196 anterior composite restorations.^7^ The calculated AFR for anterior composites was approximately 4.5%. Fillings in central incisors are most likely to fail.^7^ Another large survival study of anterior restorations was conducted in the UK using patient data.5 This included approximately 3.5 million composite restorations in cavity Classes II, IV, and V. Approximately 34% survived 15 years without re-intervention. Larger Class IV restorations survived less well than smaller Class III and Class V restorations.^5^

Randomized controlled trials are considered the gold standard in clinical research.^11^ These studies are carried out in strictly selected study populations, based on strict study protocols.^2^ But a number of aspects influence the longevity of both anterior and posterior restorations, such as patient-related factors (caries risk, bruxism, age) or clinical experience of the practitioner.^12,13,26,27^

Therefore, the aim of this study was to add information and to explore the outcome of direct anterior restorations based on a large data subset of one of the German national health insurance companies.

## Materials and Methods

As in previous studies reporting outcomes of dental treatment, a major German national health insurance company (BARMER, Berlin, Germany) provided routine claims data for analyses.^21–23^ The BARMER clientele represents roughly 10% of the German population. The study design was approved by the Ethics Committee of the Technische Universität Dresden (EK 2878072015).

A fee code represented the basic unit of information for the dental treatment. These data were analyzed for a 4-year period on a day-count basis from January 1, 2010 to December 31, 2013. Some specific German regions had to be excluded as systematical data were missing. Adult patients were selected. The patients had to be members of the insurance company for the entire 4-year observation period.

The study intervention was defined as a direct or indirect restoration (Class III and IV) involving a mesial or distal surface of anterior teeth (including incisors and canines). All restorations placed during the observation period entered the analysis. Each single restoration was under risk according to its individual time within the analysis. Deciduous teeth, as well as injured teeth after dental trauma, were excluded for methodological reasons.

Statistical analysis was performed using the Kaplan–Meier method. The primary outcome was re-intervention defined as a restoration placed on or involving the same surface. Thereafter, two secondary analyses were performed for the outcomes “crowning/partial crowning” and “extraction.” Prior to placing a crown, teeth often need a permanent restoration used as core build-ups. Therefore, crowning did not count as secondary outcome within 60 days after the restoration had been inserted.

## Results

A total of 4,826,822 restorations could be observed. Of these, 2,417,791 restorations involved mesial surfaces and 2,409,031 restorations involved distal surfaces.

**Fig 1:**
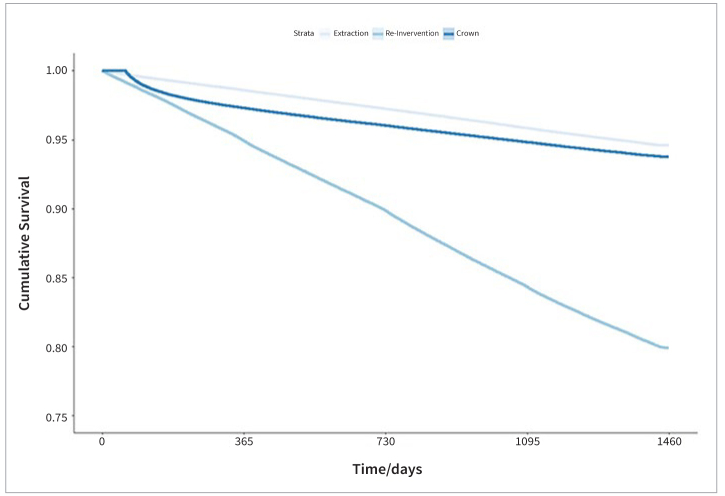
Survival curves for restorations including the mesial surface.

**Fig 2:**
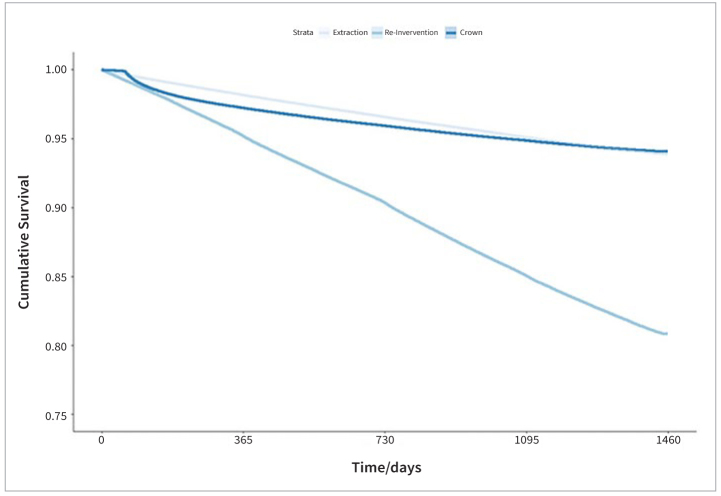
Survival curves for restorations including the distal surface.

For the primary outcome “re-intervention,” the cumulative 4-year survival rates were 79.9% for mesial (Fig 1) and 80.9% for distal restorations (Fig 2). The respective AFR for the target event “re-intervention” are between 5.0% (mesial) and 4.8% (distal). At the time of the last event, 7,700 mesial restorations and 7,658 distal restorations had been still under risk. This last event occurred after 1,454 days (3.98 years) for both mesial and distal restorations.

The cumulative 4-year survival rates for the secondary outcome “crown” were 93.8% for mesial (Fig 1) and 94.1% for distal restorations (Fig 2). The respective AFR for the target event “crown” are between 1.6% (mesial) and 1.5% (distal). 26,430 mesial restorations and 21,791 distal restorations had been still under risk at the time of the last event. This last event occurred after 1,446 days (3.96 years) at mesial restorations and after 1,444 days (3.96 years) at distal restorations.

For the secondary outcome “extraction,” the cumulative 4-year survival rates were 94.6% for mesial (Fig 1) and 93.9% for distal restorations (Fig 2). The respective AFR for the target event “extraction” was between 1.4% (mesial) and 1.5 % (distal). At the time of the last event, 22,168 mesial restorations and 22,250 distal restorations were still at risk. This last event occurred after 1,448 days (3.96 years) at mesial restorations and after 1,445 days (3.96 years) at distal restorations.

## Discussion

This study presents cumulative 4-year survival rates of anterior restored tooth surfaces between approximately 80% and 95%. That means, that every fifth restored interproximal mesial and distal surface of anterior teeth was re-intervened within 4 years. Moreover, about 6% of the teeth with restored anterior surfaces had been crowned and 5% to 6% had been extracted during the observation period.

These results are based on an exceptionally high number of cases. A total of 4.826.822 restorations were included in the analysis focusing on the primary outcome “re-intervention” and the secondary outcomes “crown” and “extraction.” The insurance database encloses the complete number of all conducted treatments from 2010 to 2013 for over 8 million members representing almost 10% of the German population at this time. These large-scale data analyses therefore have great potential for evaluating outcomes from daily practice. Similar analyses were published and are scientifically well accepted.^3,4,6,21,23^ Some important limitations have to be taken into account in order to avoid over- or misinterpretation.

Data was based on digitally submitted fee codes provided by a national health insurance company. There was no clinical documentation or any documentation of clinical findings available. Fee codes or claims data are not primarily used for scientific purposes. Within these so-called secondary data or routine data, errors or mistakes cannot be avoided completely. Because of the high number of cases, the authors assume that data errors like false tooth numbers or surfaces are balancing out to some extent. However, the data quality itself can be expected lower compared to a well-documented clinical trial. Restorations in deciduous teeth were excluded because of their different clinical performance compared to permanent teeth. If direct restorations are the consequence of trauma, they are sometimes not included in our data. This relies on the fact that the coverage of these trauma consequences is not always the responsibility of the National Health Insurance System. Sometimes, other insurances, accident parties, or other payers are bearing the costs. Therefore, they cannot always be seen in the data for methodological reasons. If a secondary restoration is replacing the first restoration after trauma, it is more likely to be seen in our data. Therefore, trauma cases cannot really be differentiated from others in the present study. In some cases, direct restorations are used as core build-ups for crowns. For that reason, a “crown” did not count as a target event when it was placed less than 60 days after the direct restoration.

The database of this study itself does not allow a differentiation between the materials used. However, there is nearly no alternative to resin composite materials in Class III and IV restorations.^7,9,19^ Therefore, it can be assumed that the vast majority of observed restorations are made from composite resin material.

Our analysis uses a surface-related approach. Class III and IV restorations in the front region are mostly involving the mesial or the distal tooth surface. Therefore, every restoration involving one of these surfaces was included. Adhesive restorations in incisors or canines have multiple shapes and surfaces. With our approach, we conclude survival rates for the wide majority of these restorations.

The Kaplan–Meier survival analysis is a statistical tool developed originally for randomized controlled trials. Nowadays, the use of the Kaplan–Meier analysis for retrospective evaluations after well-documented interventions is an established method.^23^

An interpretation of the presented results in the context of recent publications seems not easy. Studies and reviews focusing on Class III and IV restorations mostly do not use core outcomes such as re-interventions.^16^ Therefore, results are hardly comparable. Putting our results in relation to recent studies and reviews, our survival rates are lower.

A recent review by Heintze et al revealed a 10-year rate of 2.5% for secondary caries related to Class III and IV restorations.16 Of course, secondary caries is not the only cause but one of the major causes for re-intervention. Esthetics play an important role in the anterior region, so that a restoration can be renewed even in case of discoloration and esthetic discomfort.^11,12^ The studies considered in the review by Demarco et al with follow-up periods between 3 and 9 years revealed AFR from 0 to 3.7%.^11^

We expected differences between our results and those of these studies because of the well-known gap between clinical study results and results from general practice. Furthermore, insurance data do not select for patients or for dentists. This might also be seen as an advantage from a public health perspective.

Restorations observed in clinical studies are mainly first interventions at the respective surfaces. Restorations observed in digital databases may already have been re-interventions of previous restorations. Collares et al analyzed a large data set of anterior restorations placed by a network of general practitioners in the Netherlands and stated mean AFR after three (4.4%), five (4.6%), and ten years (4.6%).7 This study shows better outcomes although data was collected in a private practice setting. However, the different result is not unexpected. A direct comparison with our results is limited as significantly lower case numbers were evaluated. But the major reason for these superior results can be seen in the selection of practitioners within a private practice research network.

## Conclusion

In the present study, survival rates between restorations involving mesial and distal surfaces are quite similar. As expected, differences are small and clinically not relevant. This study was conducted in Germany based on data form a German national health insurance company. In a first view, results are therefore valid for Germany. However, techniques, materials, and procedures for direct permanent anterior restorations can be assumed to be quite comparable between Germany and other developed countries. Therefore, the authors would expect results from other developed countries to be in the same range. This statement can be underlined by the fact, that results from massive data analyses for posterior restorations from Germany and Great Britain are also in the same range.^3,18,21,23^

## Clinical relevance:

At first sight, the performance of direct anterior restorations under practice conditions is acceptable. However, a significant number of re-interventions occurred in a short-term perspective. Concerning the long-term sustainability of anterior restorations, there is still room for improvement.

## References

[ref1] Alcaraz MGR, Veitz-Keenan A, Sahrmann P, Schmidlin PR, Davis D, Iheozor-Ejiofor Z (2014). Direct composite resin fillings versus amalgam fillings for permanent or adult posterior teeth. Cochrane Database of Systematic Reviews.

[ref2] Bondemark L, Ruf S (2015). Randomized controlled trial: the gold standard or an unobtainable fallacy?. Eur J Orthod.

[ref3] Burke FJ, Lucarotti PS (2009). How long do direct restorations placed within the general dental services in England and Wales survive?. Br Dent J.

[ref4] Burke FJ, Lucarotti PS, Holder R (2005). Outcome of direct restorations placed within the general dental services in England and Wales (Part 4): influence of time and place. J Dent.

[ref5] Burke FJT, Lucarotti PSK (2018). The ultimate guide to restoration longevity in England and Wales. Part 4: resin composite restorations: time to next intervention and to extraction of the restored tooth. Br Dent J.

[ref6] Chen SC, Chueh LH, Hsiao CK, Tsai MY, Ho SC, Chiang CP (2007). An epidemiologic study of tooth retention after nonsurgical endodontic treatment in a large population in Taiwan. J Endod.

[ref7] Collares K, Opdam NJM, Laske M, Bronkhorst EM, Demarco FF, Correa MB, Huysmans M (2017). Longevity of anterior composite restorations in a general dental practice-based network. J Dent Res.

[ref8] de Kuijper MC, Cune MS, Özcan M, Gresnigt MM (2023). Clinical performance of direct composite resin versus indirect restorations on endodontically treated posterior teeth: a systematic review and meta-analysis. J Prosthet Dent.

[ref9] Demarco FF, Baldissera RA, Madruga FC, Simoes RC, Lund RG, Correa MB, Cenci MS (2013). Anterior composite restorations in clinical practice: findings from a survey with general dental practitioners. J Appl Oral Sci.

[ref10] Demarco FF, Cenci MS, Montagner AF, de Lima VP, Correa MB, Moraes RR, Opdam NJ (2023). Longevity of composite restorations is definitely not only about materials. Dent Mater.

[ref11] Demarco FF, Collares K, Coelho-de-Souza FH, Correa MB, Cenci MS, Moraes RR, Opdam NJ (2015). Anterior composite restorations: A systematic review on long-term survival and reasons for failure. Dent Mater.

[ref12] Demarco FF, Collares K, Correa MB, Cenci MS, Moraes RR, Opdam NJ (2017). Should my composite restorations last forever? Why are they failing?. Braz Oral Res.

[ref13] Ghazal TS, Cowen HJ, Caplan DJ (2018). Anterior restoration longevity among nursing facility residents: a 30-year retrospective study. Spec Care Dentist.

[ref14] Gulamali AB, Hemmings KW, Tredwin CJ, Petrie A (2011). Survival analysis of composite Dahl restorations provided to manage localised anterior tooth wear (ten-year follow-up). Br Dent J.

[ref15] Heintze SD, Loguercio AD, Hanzen TA, Reis A, Rousson V (2022). Clinical efficacy of resin-based direct posterior restorations and glass-ionomer restorations – an updated meta-analysis of clinical outcome parameters. Dent Mater.

[ref16] Heintze SD, Rousson V, Hickel R (2015). Clinical effectiveness of direct anterior restorations – a meta-analysis. Dent Mater.

[ref17] Hickel R, Roulet JF, Bayne S, Heintze SD, Mjör IA, Peters M (2007). Recommendations for conducting controlled clinical studies of dental restorative materials. Clin Oral Investig.

[ref18] Lucarotti PS, Holder RL, Burke FJ (2005). Outcome of direct restorations placed within the general dental services in England and Wales (Part 3): variation by dentist factors. J Dent.

[ref19] Montagner AF, Sande FHV, Muller C, Cenci MS, Susin AH (2018). Survival, reasons for failure and clinical characteristics of anterior/posterior composites: 8-year findings. Braz Dent J.

[ref20] Moraes RR, Cenci MS, Moura JR, Demarco FF, Loomans B, Opdam N (2022). Clinical performance of resin composite restorations. Curr Oral Health Rep.

[ref21] Raedel M, Hartmann A, Bohm S, Priess HW, Samietz S, Konstantinidis I, Walter MH (2017). Four-year outcomes of restored posterior tooth surfaces-a massive data analysis. Clin Oral Investig.

[ref22] Raedel M, Hartmann A, Bohm S, Walter MH (2015). Three-year outcomes of root canal treatment: Mining an insurance database. J Dent.

[ref23] Raedel M, Hartmann A, Priess HW, Bohm S, Samietz S, Konstantinidis I, Walter MH (2017). Re-interventions after restoring teeth-Mining an insurance database. J Dent.

[ref24] Rodolpho PADR, Rodolfo B, Collares K, Correa MB, Demarco FF, Opdam NJ (2022). Clinical performance of posterior resin composite restorations after up to 33 years. Dent Mater.

[ref25] Shah YR, Shiraguppi VL, Deosarkar BA, Shelke UR (2021). Long-term survival and reasons for failure in direct anterior composite restorations: a systematic review. J Conserv Dent.

[ref26] Stewardson D, Creanor S, Thornley P, Bigg T, Bromage C, Browne A (2012). The survival of Class V restorations in general dental practice: part 3, five-year survival. Br Dent J.

[ref27] van de Sande FH, Collares K, Correa MB, Cenci MS, Demarco FF, Opdam N (2016). Restoration survival: revisiting patients’ risk factors through a systematic literature review. Oper Dent.

